# Future Long-Term Care Needs for US Adults: Perceptions and Planning from a National Survey

**DOI:** 10.1093/geroni/igaf122.2689

**Published:** 2025-12-31

**Authors:** Ana Montoya, Erica Solway, Matthias Kirch, Dianne Singer, Sydney Strunk, J Scott Roberts, Jeffrey Kullgren, Julie Bynum

**Affiliations:** University of Michigan, Ann Arbor, Michigan, United States; University of Michigan, Ann Arbor, Michigan, United States; University of Michigan, Ann Arbor, Michigan, United States; University of Michigan, Ann Arbor, Michigan, United States; University of Michigan, Ann Arbor, Michigan, United States; University of Michigan School of Public Health, Ann Arbor, Michigan, United States; University of Michigan, Ann Arbor, Michigan, United States; University of Michigan, Ann Arbor, Michigan, United States

## Abstract

Many older adults require assistance with activities of daily living as they age. Assistance can be provided as long-term care services in the home, an assisted living facility, or a nursing home. This study aimed to understand US adults’ perceptions of and planning for future long-term care needs, using a nationally representative survey of U.S. community-dwelling adults aged 50 and older (N = 3,486). Respondents were asked about their perceived future need for long-term care services, knowledge about long-term care payors, and engagement in long-term care planning. Survey findings indicated that 42.6% reported they were likely to require long-term care in the future. However, over half were not confident that they would be able to afford to pay for long-term care services (50.9% home care, 57.7% assisted living, 62.3% nursing home care). Additionally, 61.6% incorrectly thought Medicare would cover the cost of their long-term care in a nursing home. Another 29.4% indicated Medicaid would pay for it, and nearly half (49.6%) indicated they would pay for it from their own financial resources. Half (50.1%) of respondents indicated they had not undertaken the usual steps for long-term care planning; only 27.2% reported having appointed a durable power of attorney for healthcare, and 23.7% had identified potential future caregivers. Most adults indicated they could not afford long-term care services if needed, and half of them have not taken steps to plan for such care. Educating and supporting aging US adults in planning is necessary to prepare for their future long-term care needs.

